# Gingival-Dominant Presentation of DPP-4 Inhibitor-Associated Bullous Pemphigoid: A Case Report

**DOI:** 10.7759/cureus.105274

**Published:** 2026-03-15

**Authors:** Yasuyuki Fujii, Toshiyuki Kataoka, Chika Togashi, Moe Ishikawa, Yoko Kawase-Koga

**Affiliations:** 1 Oral and Maxillofacial Surgery, Tokyo Women's Medical University, Tokyo, JPN; 2 Oral and Maxillofacial Surgery, Tokyo Women's Medical University Yachiyo Medical Center, Yachiyo, JPN

**Keywords:** anti-bp180 antibody, bullous pemphigoid, diabetes mellitus, dpp-4 inhibitor–associated bullous pemphigoid, drug-induced autoimmune disease, gingival lesions, oral manifestations

## Abstract

Bullous pemphigoid (BP) is an autoimmune subepidermal blistering disease that predominantly affects elderly individuals and has recently been associated with dipeptidyl peptidase-4 (DPP-4) inhibitor therapy. Oral involvement is uncommon, and gingival-dominant presentations are particularly rare. We report a case of DPP-4 inhibitor-associated BP presenting primarily with gingival lesions in a 76-year-old woman with type 2 diabetes mellitus treated with teneligliptin. The patient initially presented with persistent gingival white lesions and erosions, followed by the subsequent development of cutaneous bullae. Serological testing revealed elevated anti-BP180 antibody levels. Histological analysis of a skin biopsy demonstrated subepidermal blister formation with eosinophilic infiltration, and direct immunofluorescence showed linear deposition of C3, IgG, and IgA along the basement membrane zone. Teneligliptin was discontinued and treatment with topical corticosteroids, oral nicotinamide, and tetracycline-class antibiotics resulted in gradual resolution of both oral and cutaneous lesions without prolonged systemic corticosteroid therapy. Anti-BP180 antibody levels decreased in parallel with clinical improvement during long-term follow-up. This case highlights the diagnostic challenges of gingival-dominant BP and emphasizes the importance of considering DPP-4 inhibitor-associated BP in elderly patients with refractory gingival lesions, as well as the value of interdisciplinary collaboration between dentistry and dermatology.

## Introduction

Bullous pemphigoid (BP) is a chronic autoimmune blistering disease affecting the skin that predominantly affects elderly individuals and is characterized by circulating autoantibodies against hemidesmosomal proteins, most commonly BP180 and BP230 [1]. Epidemiological studies have reported an incidence of BP ranging from approximately 2.4 to 42.8 cases per million persons per year, with higher rates observed in elderly populations [2].

In recent years, dipeptidyl peptidase-4 (DPP-4) inhibitors, which are widely prescribed for the treatment of type 2 diabetes mellitus, have been increasingly recognized as a trigger for BP [3]. Epidemiological studies have demonstrated an increased risk of BP among patients with diabetes treated with DPP-4 inhibitors, and DPP-4 inhibitor-associated BP is now regarded as a distinct clinical entity [3]. Compared with conventional BP, DPP-4 inhibitor-associated BP often presents with atypical clinical features, including less inflammatory skin findings and non-bullous presentations [4]. Previous case reports have described mucous membrane pemphigoid and oral erosions in the setting of DPP-4 inhibitor use, indicating that oral involvement, while uncommon, can occur [5-7]. In contrast to the typical tense bullae observed on the skin, oral lesions may present as erosions, desquamative gingivitis, or nonspecific mucosal inflammation without obvious blister formation. In addition, histopathological findings from oral biopsy specimens may be inconclusive when epithelial detachment is not preserved, which can complicate the early diagnosis of autoimmune blistering diseases. Furthermore, gingival lesions associated with autoimmune blistering diseases may clinically resemble common periodontal conditions. Variations in gingival phenotype, such as thin or delicate gingival tissues, may influence the clinical appearance of mucosal lesions and potentially mimic inflammatory periodontal changes [8,9]. Given the widespread use of DPP-4 inhibitors among elderly patients with diabetes, recognition of this unusual presentation is particularly important for dental professionals. Here, we report a case of DPP-4 inhibitor-associated BP in a 76-year-old patient who presented with prominent gingival lesions together with cutaneous manifestations, highlighting the diagnostic challenges and the importance of interdisciplinary collaboration between dentistry and dermatology.

## Case presentation

A 76-year-old woman was referred to our department for evaluation of persistent gingival lesions. Her medical history included type 2 diabetes mellitus and postoperative status following thyroid goiter surgery. She had been treated with teneligliptin for approximately two years, along with risedronate, rosuvastatin, magnesium oxide, and shakuyakukanzoto.

One month prior to presentation, she noticed swelling of the mandibular anterior gingiva and visited her local dentist. Subsequently, white lesions developed on the maxillary anterior gingiva, which prompted referral for further examination.

**Figure 1:**
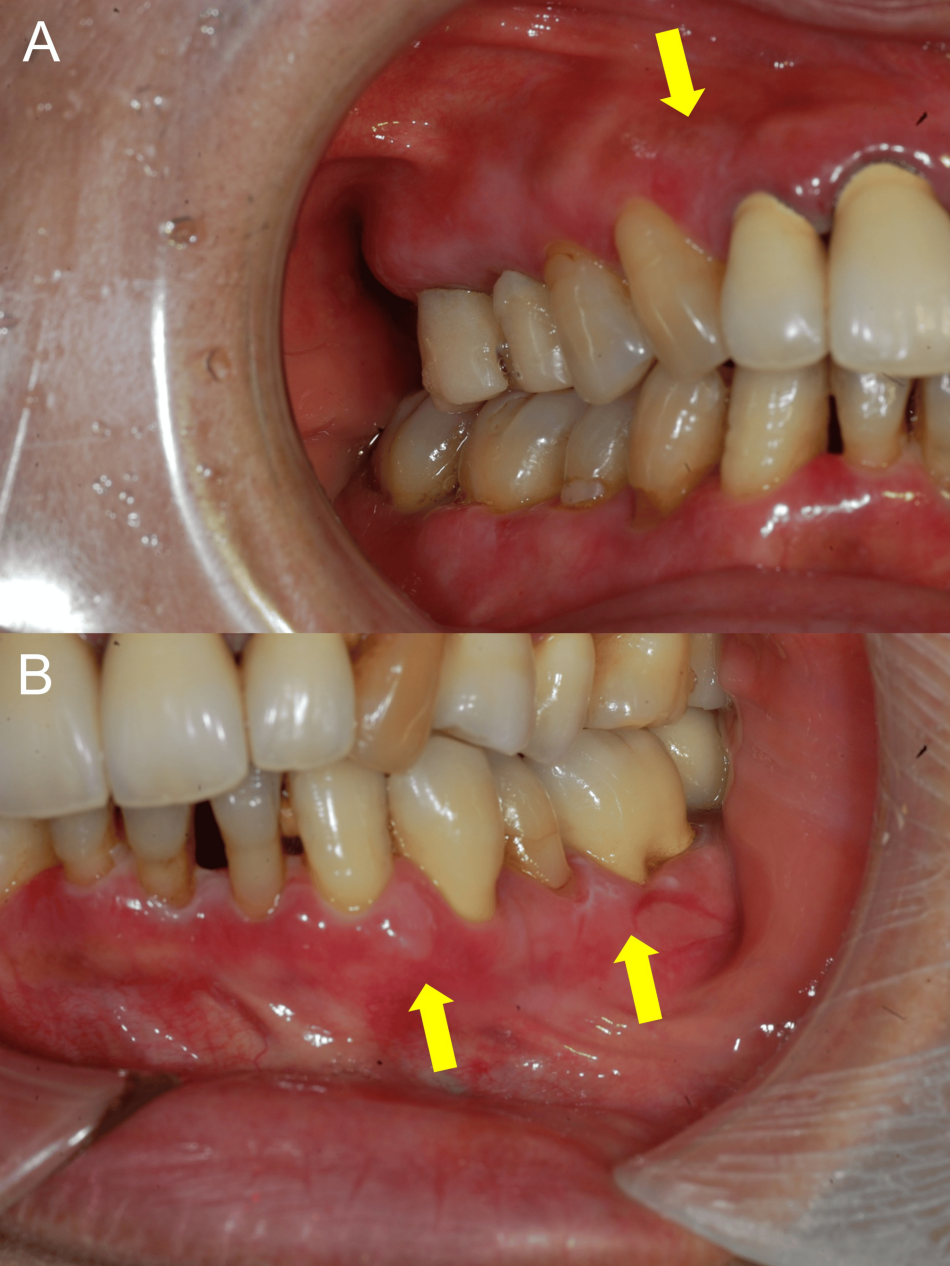
Clinical findings of the oral cavity. Intraoral photographs showing ill-defined white mucosal lesions (yellow arrows) with focal erosions on the buccal gingiva of the right maxilla (A) and right mandible (B). No intact bullae were observed, and the lesions are predominantly localized to the gingiva.

Intraoral examination revealed ill-defined white mucosal lesions with focal erosions on the buccal gingiva of the right maxilla and right mandible (Figure 1).

**Figure 2:**
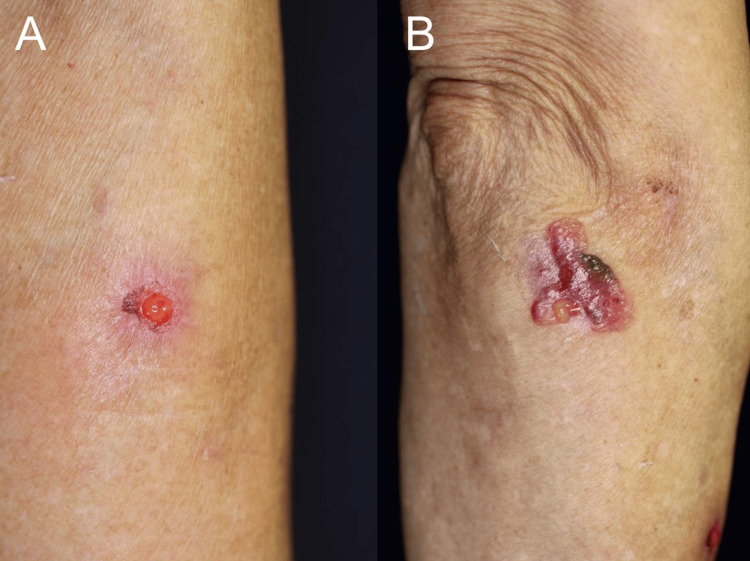
Cutaneous manifestations at initial presentation. Clinical photographs demonstrating erosions and bullae on the right forearm and erosions on the left lower extremity, accompanied by pruritus.

Nikolsky’s sign was negative on oral examination. No intact bullae were observed. Cutaneous examination revealed erosions and bullae on the right forearm and erosions on the left lower extremity, accompanied by pruritus (Figure 2).

**Figure 3:**
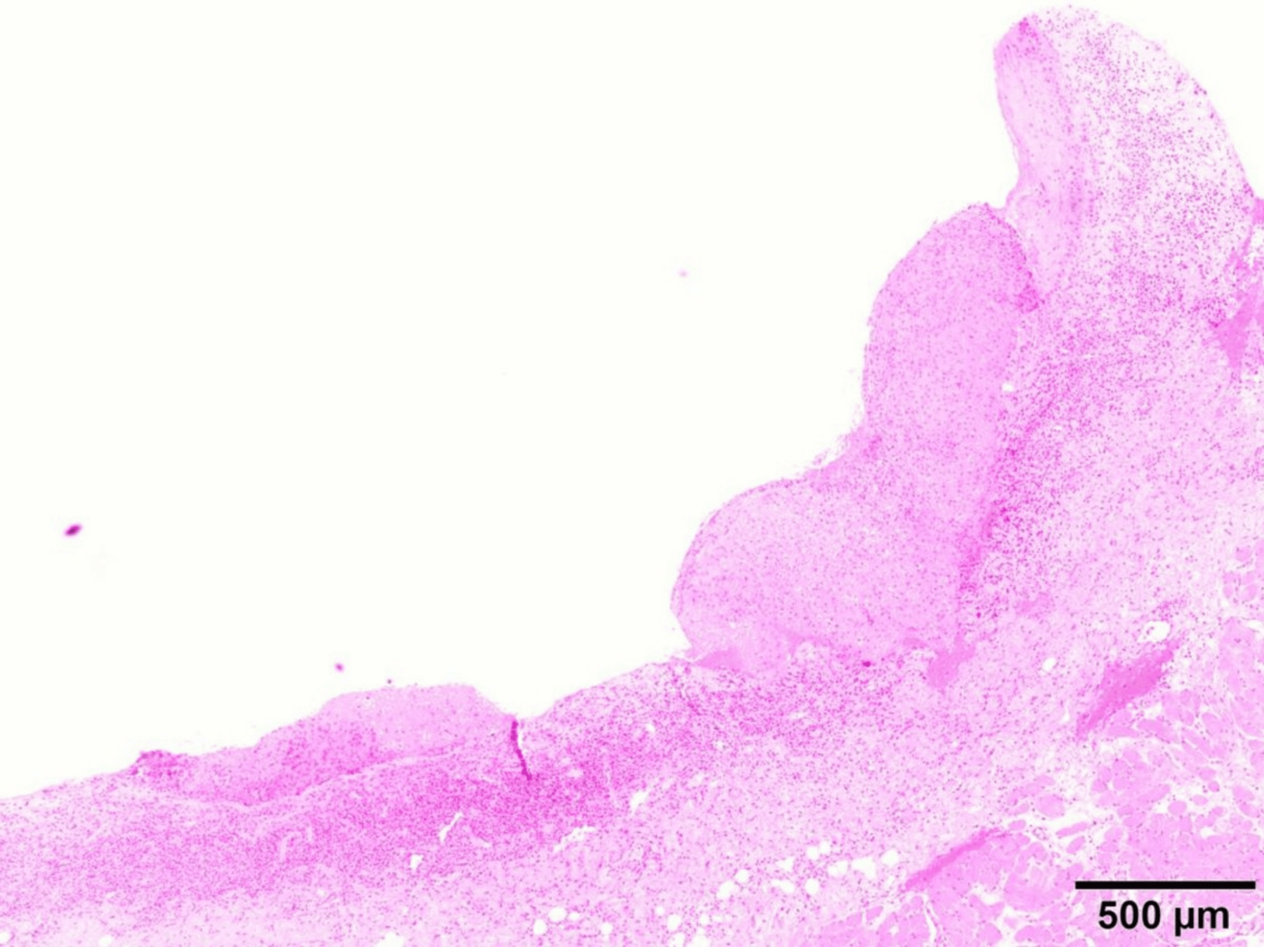
Histopathological findings of the oral biopsy. Histopathological examination of the gingival biopsy specimen from the left mandibular posterior buccal gingiva showing extensive epithelial erosion with minimal residual epithelium and no definite subepidermal blister formation (hematoxylin and eosin staining). Scale bar = 500 μm.

Serological testing demonstrated an elevated anti-BP180 antibody level of 52.5 U/mL. An oral biopsy was performed at the initial visit. Eight days later, a skin biopsy was performed by the dermatology department to further evaluate the cutaneous lesions. An oral biopsy from the left mandibular posterior buccal gingiva showed extensive erosion with minimal residual epithelium and no apparent blister formation (Figure 3).

**Figure 4:**
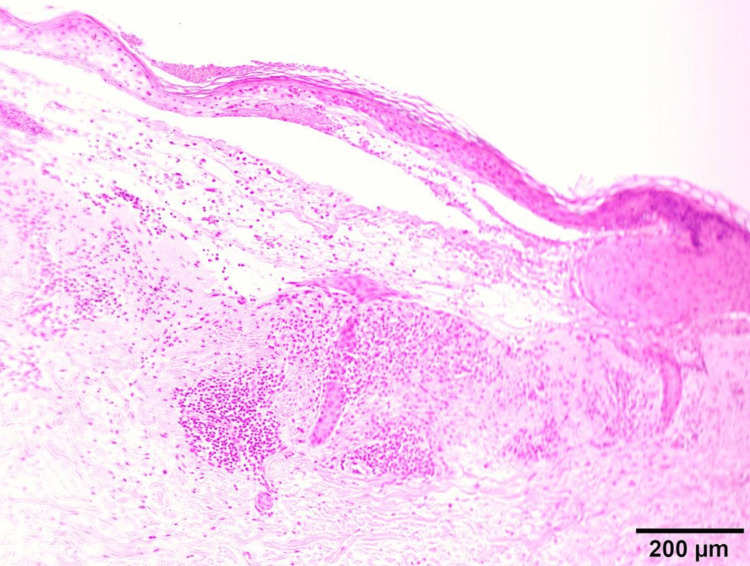
Histopathological findings of the skin biopsy. Skin biopsy specimen from the right upper limb revealing a subepidermal blister containing numerous eosinophils. Dense perivascular infiltration of eosinophils and lymphocytes is also observed in the superficial dermis (hematoxylin and eosin staining). Scale bar = 200 μm.

In contrast, a skin biopsy from the right upper limb revealed a subepidermal blister with numerous eosinophils within the blister cavity and dense perivascular infiltration of eosinophils and lymphocytes in the superficial dermis (Figure 4).

**Figure 5:**
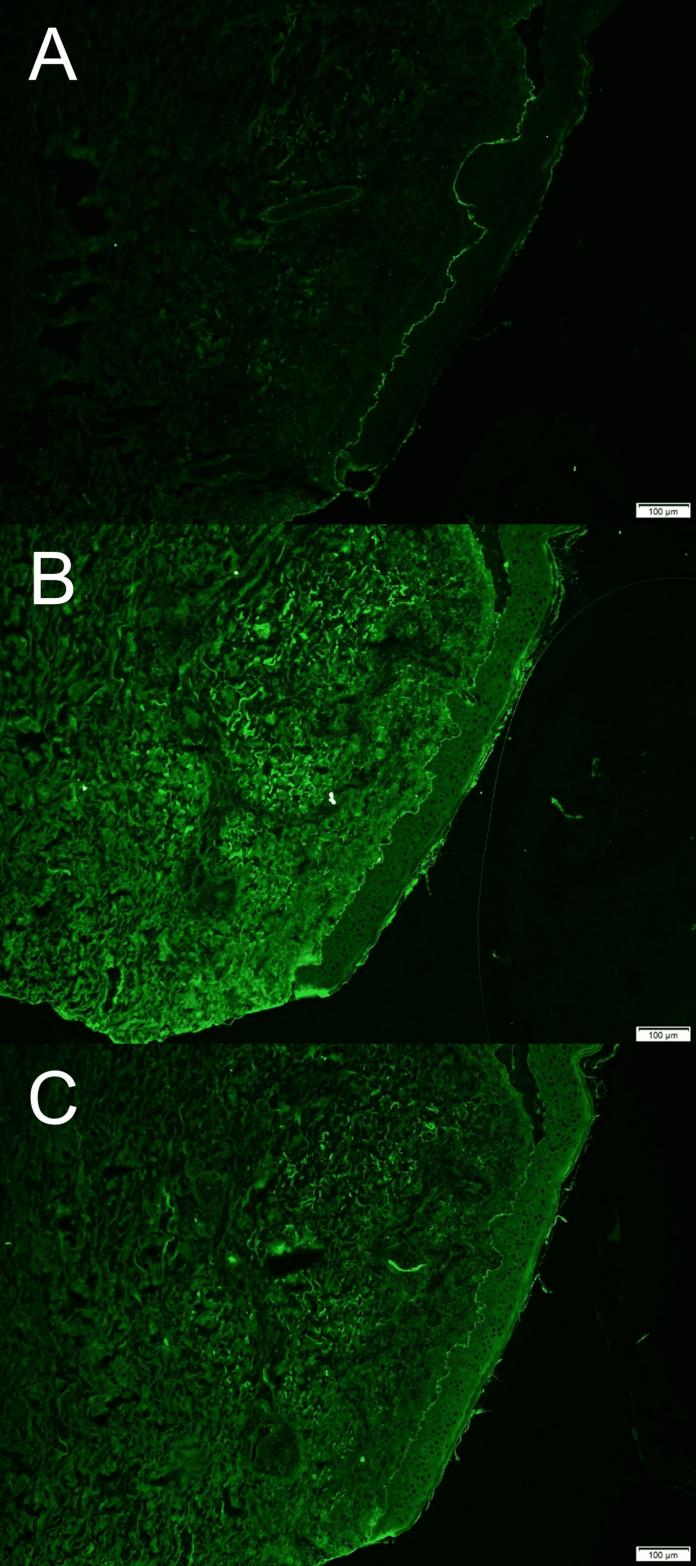
Direct immunofluorescence findings. Direct immunofluorescence microscopy of the skin specimen demonstrating linear deposition of C3 (A), IgG (B), and IgA (C) along the basement membrane zone. Scale bar = 100 μm.

Direct immunofluorescence of the skin specimen demonstrated linear deposition of C3, IgG, and IgA along the basement membrane zone, while indirect immunofluorescence was negative (Figure 5).

Based on these findings, a diagnosis of BP with both oral and cutaneous involvement was established. At the first visit, sodium azulene sulfonate gargle was prescribed as a topical anti-inflammatory and mucosal soothing agent for the oral lesions, and diflorasone diacetate ointment, a potent topical corticosteroid, was applied to the skin lesions. Dermatological evaluation and management, including skin biopsy and topical treatment, were initiated in collaboration with the Department of Dermatology at our institution. Two weeks after the initial visit, teneligliptin was discontinued and replaced with metformin for glycemic control following consultation with the patient’s primary physician.

Nine weeks after presentation, the lower extremity lesions improved; however, new bullae developed on both forearms. Oral nicotinamide (1,500 mg/day) and minocycline (100 mg/day) were initiated. Oral bullae repeatedly developed and resolved on the gingiva, alveolar mucosa, and lower lip, but completely resolved 10 months after the initial visit. During a six-year follow-up period, occasional gingival bullae occurred approximately once per year and were managed successfully with topical corticosteroids and sodium azulene sulfonate gargle.

Cutaneous symptoms showed worsening at 11 weeks, prompting an increase in the minocycline dose to 150 mg/day, and were further increased to 200 mg/day at 14 weeks due to pruritic erythema on the back. Skin lesions gradually improved, and bullae resolved nine months after the initial visit, allowing discontinuation of nicotinamide. One month later, skin lesions recurred, and nicotinamide was resumed at 750 mg/day. Due to hyperpigmentation on the dorsal fingers, a well-known adverse effect of long-term minocycline therapy, minocycline was switched to doxycycline. Serum anti-BP180 antibody levels gradually decreased in parallel with clinical improvement. The antibody level declined from 52.5 U/mL at diagnosis to 31.2 U/mL at 1 year, 17.5 U/mL at 2 years, 10.1 U/mL at three years, 6.2 U/mL at four years, 4.9 U/mL at five years, and 3.3 U/mL at six years after the initial visit. At the most recent follow-up, the patient continues doxycycline therapy (100 mg/day) and has experienced no recurrence of bullous lesions for approximately two years, with no clinically significant adverse effects observed during follow-up.

## Discussion

DPP-4 inhibitors have been identified as an important trigger for BP, particularly in older patients with diabetes, and DPP-4 inhibitor-associated BP is now considered a distinct clinical subtype [3]. The incidence of DPP-4 inhibitor-associated BP varies among studies but has been reported to be several-fold higher than that in the general population with diabetes [10,11]. Among DPP-4 inhibitors, differences in BP risk have been suggested, with some studies reporting higher associations for vildagliptin and linagliptin, although BP has been reported with multiple agents, including teneligliptin [11-13]. These findings support the concept that DPP-4 inhibitor-associated BP represents a drug-related adverse event rather than a coincidental occurrence. Given the increasing use of DPP-4 inhibitors in patients with type 2 diabetes, awareness of this potential adverse drug reaction is important, and appropriate recognition and reporting of suspected cases are essential for pharmacovigilance.

The pathomechanism underlying DPP-4 inhibitor-associated BP has not been fully elucidated; however, several hypotheses have been proposed. Previous studies have demonstrated that DPP-4 inhibitor-associated BP exhibits a distinct autoantibody profile compared with conventional BP, with reduced reactivity to the NC16A domain of BP180, suggesting altered antigen recognition [4]. DPP-4 (CD26) plays an important role in immune regulation, and its inhibition may disrupt immune homeostasis. Clinical evidence indicates that DPP-4 inhibitors can alter circulating CD4+ T-cell subsets in patients with type 2 diabetes, potentially promoting autoimmune responses [14]. In addition, DPP-4 inhibitor-associated BP does not always resolve after drug discontinuation. Shinohara et al. reported unfavorable clinical outcomes despite immediate cessation of the causative agent, suggesting that immune dysregulation may persist once triggered [15]. Collectively, these findings suggest that multiple immunological alterations induced by DPP-4 inhibition may contribute to BP development in susceptible elderly patients with diabetes.

Clinically, DPP-4 inhibitor-associated BP often exhibits less inflammatory skin manifestations and may present with non-bullous or atypical lesions compared with conventional BP [4]. In the present case, the patient initially presented with gingival swelling and whitish mucosal lesions without obvious blister formation, and the Nikolsky sign was negative. These findings contributed to the diagnostic difficulty in the early stage. BP presenting primarily as gingival or mucosal lesions may mimic periodontal disease or other inflammatory oral conditions, leading to delayed diagnosis. In the early stage, gingival swelling, erythema, or erosion may resemble common dental conditions such as plaque-induced gingivitis or periodontal inflammation. In addition, desquamative gingivitis associated with autoimmune mucocutaneous diseases should also be considered in the differential diagnosis. Conditions such as mucous membrane pemphigoid, pemphigus vulgaris, and erosive oral lichen planus may present with similar gingival erythema or mucosal desquamation. Therefore, careful clinical evaluation combined with histopathological and immunological examinations is essential for accurate diagnosis. Histopathological findings from the gingival biopsy were nonspecific, which is consistent with a previous report indicating that oral biopsies alone may be insufficient for definitive diagnosis [16]. Serum anti-BP180 antibody levels were elevated at diagnosis and gradually decreased in parallel with clinical improvement, supporting previous findings that anti-BP180 antibody titers correlate with disease activity [17]. Discontinuation of the suspected causative drug and adjunctive therapy with oral nicotinamide and tetracycline-class antibiotics contributed to long-term disease control.

Discontinuation of the suspected causative drug is considered a cornerstone of management in drug-induced BP [3,18]. In the present case, teneligliptin was discontinued in consultation with the patient’s primary physician and replaced with metformin to maintain glycemic control. In addition, nicotinamide combined with tetracycline-class antibiotics has been reported as an effective corticosteroid-sparing therapeutic option, particularly in elderly patients or those with multiple comorbidities [19]. In the present case, withdrawal of teneligliptin and combination therapy resulted in satisfactory control of both oral and cutaneous lesions without the need for prolonged systemic corticosteroid therapy.

## Conclusions

From a dental perspective, this case underscores the importance of considering autoimmune blistering diseases, including DPP-4 inhibitor-associated BP, in the differential diagnosis of refractory gingival lesions in elderly patients with diabetes. Oral mucosal involvement in BP is generally uncommon, and gingival-dominant presentations are particularly rare. As a result, gingival lesions may be misdiagnosed as periodontal disease or other inflammatory oral conditions. The present case is notable because the patient initially presented with gingival lesions that mimicked common periodontal conditions, illustrating the diagnostic challenges that may arise in dental practice. When persistent or atypical gingival lesions are encountered, early biopsy and timely referral to dermatology should be considered to facilitate accurate diagnosis. Early recognition and prompt interdisciplinary collaboration with dermatology may facilitate accurate diagnosis and appropriate management. However, alternative factors, such as concomitant medications or age-related immune dysregulation, may also contribute to the development of BP, and observations from a single case cannot establish the incidence or magnitude of risk associated with DPP-4 inhibitor use.
